# Supplementary material for: Precision engineering of the probiotic Escherichia coli Nissle 1917 with prime editing
Source: Appl Environ Microbiol. 2025 Jan 31;91(2):e00031-25. doi: 10.1128/aem.00031-25 (PMC11837520; doi:10.1128/aem.00031-25)
Supplement: Supplemental material — Tables S1 to S6; Figures S1 to S11. [file aem.00031-25-s0002.pdf]

Supplemental Material

Precision engineering of the probiotic *Escherichia coli* Nissle 1917 with prime editing

Pei-Ru Chen [1], Ying Wei [1], Xin Li [1], Hai-Yan Yu [2], Shu-Guang Wang [1,3,4], Xian-Zheng Yuan [1,3], and Peng-Fei Xia [1,*]

[1] School of Environmental Science and Engineering, Shandong University, Qingdao 266237, China

[2] State Key Laboratory of Microbial Technology, Shandong University, Qingdao, 266237 China

[3] Sino-French Research Institute for Ecology and Environment, Shandong University, Qingdao 266237, China

[4] Weihai Research Institute of Industrial Technology, Shandong University, Weihai 264209, China

* Correspondence:

Peng-Fei Xia

School of Environmental Science and Engineering, Shandong University, Qingdao 266237, China.

Email: pfxia@sdu.edu.cn

Table S1. Plasmids used in this study.

Name	Description	Source
pBR322	*colE1 ori, bla, tetA*	Lab stock
pRE118	*R6K γ* ori, *sacB*, *kanR*	Lab stock
pKD46	*oriR101*, *bla*	Lab stock (1)
pBBR1MCS-5	*pBBR1* ori, *GmR*	Lab stock (2)
pAM4787	*ColE1 ori*, partial sequence from pANS	Addgene #120088 (3)
pCMV-PE2-P2A-GFP	Cas9 (H840A)-M-MLVrt (D200N, T306K, W313F, T330P, L603W)	Addgene #132776 (4)
pWY	pBBR1MCS-5, *lacI*-P_{trc}, *dcas9*, *PmCDA1*, *ugi*	Lab stock (2)
pmCherry	pBBR1MCS-5, *lacI*-P_{trc}, *mCherry*	Lab stock (2)
pBeSpRY	*oriR101*, *bla*, *trc* promoter, *dSpRY-PmCDA1-ugi*	Lab stock (5)
pBeCas9	*oriR101*, *bla*, *trc* promoter, *dCas9-PmCDA1-ugi*	Lab stock (5)
pPegRNA01	pUC57, pegRNA01	This study
pPegRNA02	pUC57, pegRNA02	This study
pPegRNA03	pUC57, pegRNA03	This study
pPegRNA04	pUC57, pegRNA04	This study
pPegRNA05	pUC57, pegRNA05	This study
pPegRNA06	pUC57, pegRNA06	This study
pPegRNA07	pUC57, pegRNA07	This study
pgRNA-stop	pUC57, gRNA01	This study
pgRNA-start	pUC57, gRNA02	This study
pPE.H	PE.H (*ncas9* from pCMV-PE2-P2A-GFP fused with *E. coli* codon-optimized RT), *lacI*-P_{trc}, *GmR*, *oriR101*	This study
pPE.S	PE.S (*ncas9* from *S. pyogenes* with native sequence fused with *E. coli* codon-optimized RT), *lacI*-P_{trc}, *GmR*, *oriR101*	This study

pRC01	pPE.H, pegRNA01	This study
pRC02	pPE.H, pegRNA02	This study
pRC03	pPE.H, pegRNA03	This study
pRC04	pPE.S, pegRNA01	This study
pRC05	pPE.S, pegRNA02	This study
pRC06	pPE.S, pegRNA03	This study
pRC07	pPE.H, pegRNA04	This study
pRC08	pPE.H, pegRNA05	This study
pRC09	pPE.H, pegRNA06	This study
pRC10	pPE.H, pegRNA07	This study
placI-mCherry01	pRC02, PE.H::*mcherry, lacI*-P_{trc}, *GmR*, *oriR101*	This study
placI-mCherry02	placI-mCherry01, *oriR101*::*pMB* ori*, sacB*	This study
pRED	pmCherry, *GmR*::$glnA^{m}$	This study
pGREEN	pBR322, *gfp*, $argH^{m}$	This study
pBeCas9-iSTOP	pBeCas9, gRNA-stop	This study
pBeSpRY-XSTART	pBeSpRY, gRNA-start	This study

Table S2. Sequences of codon-optimized RT and *glnA*^{m} and *argH*^{m}.

Gene	Sequence
codon-optimized RT	ACCCTGAACATCGAAGACGAATACCGTCTGCACGAAACCTCTAAA<br>GAACCGGACGTTTCTCTGGGTTCTACCTGGCTGTCTGACTTCCCG<br>CAGGCTTGGGCTGAAACCGGTGGTATGGGTCTGGCTGTTCGTCA<br>GGCTCCGCTGATCATCCCGCTGAAAGCTACCTCTACCCCGGTTTC<br>TATCAAACAGTACCCGATGTCTCAGGAAGCTCGTCTGGGTATCAA<br>ACCGCACATCCAGCGTCTGCTGGACCAGGGTATCCTGGTTCCGT<br>GCCAGTCTCCGTGGAACACCCCGCTGCTGCCGGTTAAAAAACCG<br>GGTACCAACGACTACCGTCCGGTTCAGGACCTGCGTGAAGTTAAC<br>AAACGTGTTGAAGACATCCACCCGACCGTTCCGAACCCGTACAAC<br>CTGCTGTCTGGTCTGCCGCCGTCTCACCAGTGGTACACCGTTCTG<br>GACCTGAAAGACGCTTTCTTCTGCCTGCGTCTGCACCCGACCTCT<br>CAGCCGCTGTTCGCTTTCGAATGGCGTGACCCGGAAATGGGTATC<br>TCTGGTCAGCTGACCTGGACCCGTCTGCCGCAGGGTTTCAAAAAC<br>TCTCCGACCCTGTTCAACGAAGCTCTGCACCGTGACCTGGCTGAC<br>TTCCGTATCCAGCACCCGGACCTGATCCTGCTGCAGTACGTTGAC<br>GACCTGCTGCTGGCTGCTACCTCTGAACTGGACTGCCAGCAGGG<br>TACCCGTGCTCTGCTGCAGACCCTGGGTAACCTGGGTTACCGTGC<br>TTCTGCTAAAAAAGCTCAGATCTGCCAGAAACAGGTTAAATACCTG<br>GGTTACCTGCTGAAAGAAGGTCAGCGTTGGCTGACCGAAGCTCGT<br>AAAGAAACCGTTATGGGTCAGCCGACCCCGAAAACCCCGCGTCA<br>GCTGCGTGAATTCCTGGGTAAAGCTGGTTTCTGCCGTCTGTTCAT<br>CCCGGGTTTCGCTGAAATGGCTGCTCCGCTGTACCCGCTGACCAA<br>ACCGGGTACCCTGTTCAACTGGGGTCCGGACCAGCAGAAAGCTT<br>ACCAGGAAATCAAACAGGCTCTGCTGACCGCTCCGGCTCTGGGT<br>CTGCCGGACCTGACCAAACCGTTCGAACTGTTCGTTGACGAAAAA<br>CAGGGTTACGCTAAAGGTGTTCTGACCCAGAAACTGGGTCCGTGG<br>CGTCGTCCGGTTGCTTACCTGTCTAAAAAACTGGACCCGGTTGCT<br>GCTGGTTGGCCGCCGTGCCTGCGTATGGTTGCTGCTATCGCTGTT<br>CTGACCAAAGACGCTGGTAAACTGACCATGGGTCAGCCGCTGGTT<br>ATCCTGGCTCCGCACGCTGTTGAAGCTCTGGTTAAACAGCCGCCG<br>GACCGTTGGCTGTCTAACGCTCGTATGACCCACTACCAGGCTCTG<br>CTGCTGGACACCGACCGTGTTCAGTTCGGTCCGGTTGTTGCTCTG<br>AACCCGGCTACCCTGCTGCCGCTGCCGGAAGAAGGTCTGCAGCA<br>CAACTGCCTGGACATCCTGGCTGAAGCTCACGGTACCCGTCCGG<br>ACCTGACCGACCAGCCGCTGCCGGACGCTGACCACACCTGGTAC<br>ACCGACGGTTCTTCTCTGCTGCAGGAAGGTCAGCGTAAAGCTGGT<br>GCTGCTGTTACCACCGAAACCGAAGTTATCTGGGCTAAAGCTCTG<br>CCGGCTGGTACCTCTGCTCAGCGTGCTGAACTGATCGCTCTGACC<br>CAGGCTCTGAAAATGGCTGAAGGTAAAAAACTGAACGTTTACACC<br>GACTCTCGTTACGCTTTCGCTACCGCTCACATCCACGGTGAAATC<br>TACCGTCGTCGTGGTTGGCTGACCTCTGAAGGTAAAGAAATCAAA<br>AACAAAGACGAAATCCTGGCTCTGCTGAAAGCTCTGTTCCTGCCG<br>AAACGTCTGTCTATCATCCACTGCCCGGGTCACCAGAAAGGTCAC<br>TCTGCTGAAGCTCGTGGTAACCGTATGGCTGACCAGGCTGCTCGT<br>AAAGCTGCTATCACCGAAACCCCGGACACCTCTACCCTGCTGATC

	GAAAACTCTTCTCCGTCTGGTGGTTCTAAACGTACCGCTGACGGT<br>TCTGAATTCGAATGA
glnA^{m}	ATGTCTGCTGAACACGTTCTGACCATGCTGAACGAACACGAAGTT<br>AAATTCGTTGACCTGCGTTTCACCGACACCAAAGGTAAAGAACAG<br>CACGTTACCATCCCGGCTCACCAGGTTAACGCTGAATTCTTCGAA<br>GAAGGTAAAATGTTCGACGGTTCTTCTATCGGTGGTTGGAAAGGT<br>ATCAACGAATCTGACATGGTTCTGATGCCGGACGCTTCTACCGCT<br>GTTATCGACCCGTTCTTCGCTGACTCTACCCTGATCATCCGTTGC<br>GACATCCTGGAACCGGGTACCCTGCAGGGTTACGACCGTGACCC<br>GCGTTCTATCGCTAAACGTGCTGAAGACTACCTGCGTTCTACCGG<br>TATCGCTGACACCGTTCTGTTCGGTCCGGAACCGGAATTCTTCCT<br>GTTCGACGACATCCGTTTCGGTTCTTCTATCTCTGGTTCTCACGTT<br>GCTATCGACGACATCGAAGGTGCTTGGAACTCTTCTACCCAGTAC<br>GAAGGTGGTAACAAAGGTCACCGTCCGGCTGTTAAAGGTGGTTAC<br>TTCCCGGTTCCGCCGGTTGACTCTGCTCAGGACATCCGTTCTGAA<br>ATGTGCCTGGTTATGGAACAGATGGGTCTGGTTGTTGAAGCTCAC<br>CACCACGAAGTTGCTACCGCTGGTCAGAACGAAGTTGCTACCCGT<br>TTCAACACCATGACCAAAAAAGCTGACGAAATCCAGATCTACAAAT<br>ACGTTGTTCACAACGTTGCTCACCGTTTCGGTAAAACCGCTACCTT<br>CATGCCGAAACCGATGTTCGGTGACAACGGTTCTGGTATGCACTG<br>CCACATGTCTCTGTCTAAAAACGGTGTTAACCTGTTCGCTGGTGAC<br>AAATACGCTGGTCTGTCTGAACAGGCTCTGTACTACATCGGTGGT<br>GTTATCAAACACGCTAAAGCTATCAACGCTCTGGCTAACCCGACC<br>ACCAACTCTTACAAACGTCTGGTTCCGGGTTACGAAGCTCCGGTT<br>ATGCTGGCTTACTCTGCTCGTAACCGTTCTGCTTCTATCCGTATCC<br>CGGTTGTTTCTTCTCCGAAAGCTCGTCGTATCGAAGTTCGTTTCCC<br>GGACCCGGCTGCTAACCCGTACCTGTGCTTCGCTGCTCTGCTGAT<br>GGCTGGTCTGGACGGTATCAAAAACAAAATCCACCCGGGTGAAGC<br>TATGGACAAAAACCTGTACGACCTGCCGCCGGAAGAAGCTAAAGA<br>AATCCCGCAGGTTGCTGGTTCTCTGGAAGAAGCTCTGAACGAACT<br>GGACCTGGACCGTGAATTCCTGAAAGCTGGTGGTGTTTTCACCGA<br>CGAAGCTATCGACGCTTACATCGCTCTGCGTCGTGAAGAAGACGA<br>CCGTGTTCGTATGACCCCGCACCCGGTTGAATTCGAACTGTACTA<br>CTCTGTTTAA
argH^{m}	ATGGCTCTGTGGGGTGGTCGTTTCACCCAGGCTGCTGACCAGCG<br>TTTCAAACAGTTCAACGACTCTCTGCGTTTCGACTACCGTCTGGCT<br>GAACAGGACATCGTTGGTTCTGTTGCTTGGTCTAAAGCTCTGGTTA<br>CCGTTGGTGTTCTGACCGCTGAAGAACAGGCTCAGCTGGAAGAA<br>GCTCTGAACGTTCTGCTGGAAGACGTTCGTGCTCGTCCGCAGCAG<br>ATCCTGGAATCTGACGCTGAAGACATCCACTCTTGGGTTGAAGGT<br>AAACTGATCGACAAAGTTGGTCAGCTGGGTAAAAAACTGCACACC<br>GGTCGTTCTCGTAACGACCAGGTTGCTACCGACCTGAAACTGTGG<br>TGCAAAGACACCGTTTCTGAACTGCTGACCGCTAACCGTCAGCTG<br>CAGTCTGCTCTGGTTGAAACCGCTCAGAACAACCAGGACGCTGTT<br>ATGCCGGGTTACACCCACCTGCAGCGTGCTCAGCCGGTTACCTTC<br>GCTCACTGGTGCCTGGCTTACGTTGAAATGCTGGCTCGTGACGAA

	TCTCGTCTGCAGGACGCTCTGAAACGTCTGGACGTTTCTCCGCTG<br>GGTTGCGGTGCTCTGGCTGGTACCGCTTACGAAATCGACCGTGAA<br>CAGCTGGCTGGTTGGCTGGGTTTCGCTTCTGCTACCCGTAACTCT<br>CTGGACTCTGTTTCTGACCGTGACCACGTTCTGGAACTGCTGTCT<br>GCTGCTGCTATCGGTATGGTTCACCTGTCTCGTTTCGCTGAAGAC<br>CTGATCTTCTTCAACACCGGTGAAGCTGGTTTCGTTGAACTGTCTG<br>ACCGTGTTACCTCTGGTTCTTCTCTGATGCCGCAGAAAAAAAACCC<br>GGACGCTCTGGAACTGATCCGTGGTAAATGCGGTCGTGTTCAGG<br>GTGCTCTGACCGGTATGATGATGACCCTGAAAGGTCTGCCGCTGG<br>CTTACAACAAAGACATGCAGGAAGACAAAGAAGGTCTGTTCGACG<br>CTCTGGACACCTGGCTGGACTGCCTGCACATGGCTGCTCTGGTTC<br>TGGACGGTATCCAGGTTAAACGTCCGCGTTGCCAGGAAGCTGCTC<br>AGCAGGGTTACGCTAACGCTACCGAACTGGCTGACTACCTGGTTG<br>CTAAAGGTGTTCCGTTCCGTGAAGCTCACCACATCGTTGGTGAAG<br>CTGTTGTTGAAGCTATCCGTCAGGGTAAAGCTCTGGAAGACCTGC<br>CGCTGTCTGAACTGCAGAAATTCTCTCAGGTTATCGGTGAAGACG<br>TTTACCCGATCCTGTCTCTGCAGTCTTGCCTGGACAAACGTGCTG<br>CTAAAGGTGGTGTTTCTCCGCAGCAGGTTGCTCAGGCTATCGCTT<br>TCGCTCAGGCTCGTCTGGGTTAA

Table S3. pegRNA and gRNA sequences used in this study.

pegRNA (gRNA)	Target	PAM	spacer	PBS	RTT
pegRNA01	*glnA*	AGG	CTTGAACCTGG CACCCTGCA	AGGGTGC CAGGTT	GTCATAGCACTTGC
pegRNA02	*glnA*	AGG	CTTGAACCTGG CACCCTGCA	AGGGTGC CAGGTT	GGTCATAGCTTGC
pegRNA03	*glnA*	AGG	CTTGAACCTGG CACCCTGCA	AGGGTGC CAGGTT	GTCATAGCACTAC
pegRNA04	*glnA*	TGG	TATTCGTTCTGA AATGTGTC	ACATTTCA GAACG	TCCATCACCTAGAC
pegRNA05	*glnA*	TGG	TATTCGTTCTGA AATGTGTC	ACATTTCA GAACG	TTCCATCACAGAC
pegRNA06	*glnA*	TGG	TATTCGTTCTGA AATGTGTC	ACATTTCA GAACG	TCCATCTACAGAC
pegRNA07	*argH*	CGG	TACCGTTAGCG AGTTGCTGA	GCAACTC GCTAAC	CGGTTAGCATCGTG GCGTCA
gRNA-stop	*glnA*	TGG	GAGCAGGCACT GTACTACAT	-	-
gRNA-start	*glnA*	ATT	CCAGGAGAGTT AAAGTATGT	-	-

Table S4. Strains used in this study.

Name	Features [a]	Source
E. coli Nissle 1917	Wild type strain (EcN).	From Ho lab
PR01	EcN, DNA insertion at the locus 299 in *glnA*.	This study
PR02	EcN, DNA deletion at the locus 299 in *glnA*.	This study
PR03	EcN, DNA substitution at the loci 295, 297 and 298 in *glnA*.	This study
PR07	EcN, DNA insertion at the locus 594 in *glnA*.	This study
PR08	EcN, DNA deletion at the locus 594 in *glnA*.	This study
PR09	EcN, DNA substitution at the loci 595 and 596 in *glnA*.	This study
PR10	EcN, DNA insertion at the locus 391 in *argH*.	This study
PR13	PR10, DNA insertion at the locus 299 in *glnA*.	This study
PR17	EcN, early STOP codon insertion in *glnA*.	This study
PR18	EcN, START codon exclusion in *glnA*.	This study

[a.] Locus is counted from the first nucleotide of a specific gene.

Table S5. PR10/MG1655 abundance prediction by TIDER.

**PR10 /MG1655 (%)**	**PR10/MG1655 Abundance predicted by TIDER (%)**				**Mean** [b]	**SD** [c]
	Exp-1 [a]	Exp-2	Exp-3	Exp-4		
0.0	0.0	0.0	0.0	0.0	0.0	0.0
1.0	0.1	0.1	0.1	0.1	0.1	0.0
5.0	0.5	0.3	1.2	0.3	0.6	0.4
10.0	9.1	6.9	9.1	7.9	8.3	0.9
20.0	8.6	9.8	9.9	7.6	9.0	0.9
50.0	51.0	48.5	50.9	52.9	50.8	1.6
100.0	100.0	100.0	100.0	100.0	100.0	0.0

[a.] Analysis of an individual sequencing result of one defined strain ratio.

[b.] The average of four abundance results.

[c.] Standard deviation of four abundance results.

Table S6. Primers used in this study.

Primer	Sequence
**Primers for In-Fusion DNA assembly**	
XIA-PRC-107	GACCGCCTACGGCGGCTGCGAAGGATCTAGGTGAAGATCC
XIA-PRC-108	CGGGGAGGCAGACAAGGTATCCCTTTGATATGTAACGGTG
XIA-PRC-109	GGATCTTCACCTAGATCCTTCGCAGCCGCCGTAGGCGGTC
XIA-PRC-110	GCCGATGCTGTACTTCTTGTCCATCATGGTCTGTTTCCTGTGTG
XIA-PRC-111	CACCGTTACATATCAAAGGGATACCTTGTCTGCCTCCCCG
XIA-PRC-112	GACGGCAGCGAATTCGAGTGACCATGCGAGAGTAGGGAACT
XIA-PRC-113	CATTTGAGAAGCACACGGTCACAGGAAACAGCTATGACCG
XIA-PRC-114	TTGACTACCGGAAGCAGTGTTCTAGATTGTAAAACGACGGCCAGTC
XIA-PRC-115	AGTTCCCTACTCTCGCATGGTCACTCGAATTCGCTGCCGTC
XIA-PRC-116	CACACAGGAAACAGACCATGATGGACAAGAAGTACAGCATCGGC
XIA-PRC-132	CACACAGGAAACAGACCATGATGGATAAGAAATACTCAATAGG
XIA-PRC-133	TCCCAGCTGAGACAAATCAATGCGTGTTTCATAAAGACCAGTGAT
XIA-PRC-134	TCTTTATGAAACACGCATTGATTTGTCTCAGCTGGGAGGTGACTC
XIA-PRC-135	CCTATTGAGTATTTCTTATCCATCATGGTCTGTTTCCTGTGTG
XIA-PRC-136	TGTCGATGCCATTGTTCCAC
XIA-PRC-137	GTGGAACAATGGCATCGACA
XIA-PRC-142	ACGGTTCTGAATTCGAATGACCATGCGAGAGTAGGGAACT
XIA-PRC-143	AGTTCCCTACTCTCGCATGGTCATTCGAATTCAGAACCGT
XIA-PRC-160	GTCACCTCCCAGCTGAGACAGGTCGATCCGTGTCTCGTAC
XIA-PRC-161	GTACGAGACACGGATCGACCTGTCTCAGCTGGGAGGTGAC
XIA-PRC-162	AATGCCGATATCCTATTGGCGGCTCTTGTATCTATCAGTG
XIA-PRC-163	GTCTGACGCTCAGTGGAACG

XIA-PRC-164	CCCTTAACGTGAGTTTTCGT
XIA-PRC-165	GCAGGTATATGTGATGGGTTGAACATGTGAGCAAAAGGCC
XIA-PRC-166	GGCCTTTTGCTCACATGTTCAACCCATCACATATACCTGC
XIA-PRC-167	CACTGATAGATACAAGAGCCGCCAATAGGATATCGGCATT
XIA-PRC-168	CACACAGGAAACAGACCATGATGGTGAGCAAGGGCGAGGAGGAT
XIA-PRC-169	AGTTCCCTACTCTCGCATGGCTACTTGTACAGCTCGTCCA
XIA-PRC-170	TGGACGAGCTGTACAAGTAGCCATGCGAGAGTAGGGAACT
XIA-PRC-171	ATCCTCCTCGCCCTTGCTCACCATCATGGTCTGTTTCCTGTGTG
XIA-PRC-178	CCTTGTCGCCTTGCGTATAACACGACGTTGTAAAACGACG
XIA-PRC-179	CGTTGAATCGGGATATGCAGCACAGGAAACAGCTATGACC
XIA-PRC-180	CGTCGTTTTACAACGTCGTGTTATACGCAAGGCGACAAGG
XIA-PRC-181	GGTCATAGCTGTTTCCTGTGCTGCATATCCCGATTCAACG
XIA-PRC-182	GAACAGGCACTGTACTACATGTTTTAGAGCTAGAAATAGC
XIA-PRC-183	ATGTAGTACAGTGCCTGTTCGCTAGCATTATACCTAGGAC
XIA-PRC-194	AACGACAGGAGCACGATCATCACGACGTTGTAAAACGACG
XIA-PRC-195	CTGTTCGGTTCGTAAGCTGTCACAGGAAACAGCTATGACC
XIA-PRC-196	GAGCATTGGCCACATGTGCTCCCTTAACGTGAGTTTTCGT
XIA-PRC-197	CGTCGTTTTACAACGTCGTGATGATCGTGCTCCTGTCGTT
XIA-PRC-198	GGTCATAGCTGTTTCCTGTGACAGCTTACGAACCGAACAG
XIA-PRC-199	ACGAAAACTCACGTTAAGGGAGCACATGTGGCCAATGCTC
XIA-PRC-298	GACTGGCCGTCGTTTTACAATCTAGAACACTGCTTCCGGTAGTCAA
XIA-PRC-299	CGGTCATAGCTGTTTCCTGTGACCGTGTGCTTCTCAAATG
**Primers for colony PCR and sequencing**	
XIA-PRC-129	GCGCTTCACCGATACCAAAG
XIA-PRC-130	GAACACGTACTGACGATGCT

XIA-PRC-131	TTCGCCCGGATGGATCTTGT
XIA-PRC-175	ACGACTCACTGCGCTTTGAT
XIA-PRC-176	CGATTTCATAGGCCGTTCCT
XIA-PRC-177	CTGAACGTGCTGCTGGAAGA
XIA-PRC-186	GCTCTGGTGATTCAGGACTC
XIA-PRC-187	CAATGGTTCGTTCTCATGGC
XIA-PRC-200	TGACAAAAGCGGTTCGGCAG
XIA-PRC-201	TTCCAGTCGGGAAACCTGTC
XIA-PRC-202	CCAGACTTTACGAAACACGG
XIA-PRC-203	ACCGGTTTCGAATGCGTGGA
XIA-PRC-300	CAATCGAGGAGCCGTCAAAC
XIA-PRC-301	GAACGTACCGGATTGTTGGA
XIA-PRC-302	CCACCTTCATATTGGGTGGA
113	CATTTGAGAAGCACACGGTCACAGGAAACAGCTATGACCG
114	TTGACTACCGGAAGCAGTGTTCTAGATTGTAAAACGACGGCCAGTC
253	TGGTGTTGAAACGGGTAGCA
254	GCTACCCGTAACTCTCTGGA
M13F	GTAAAACGACGGCCAGT
M13R	GTCATAGCTGTTTCCTG

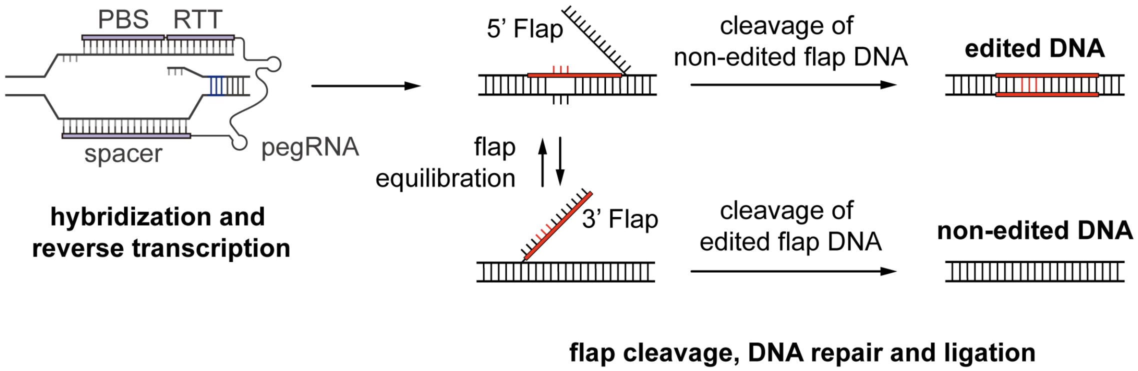

Figure S1. Schematic illustration of prime editing mechanism. PBS hybridizes to the exposed 3’ end of the nicked DNA strand, and the reverse transcription of RTT is initiated to embed the designed edits in the 3’ flap. The intended edits will be incorporated into the genome via DNA repair and 3’ flap ligation when the 3’ flap is retained and the 5’ flap is cleaved during the flap equilibration process. PBS, primer binding site; RTT, reverse transcription template.

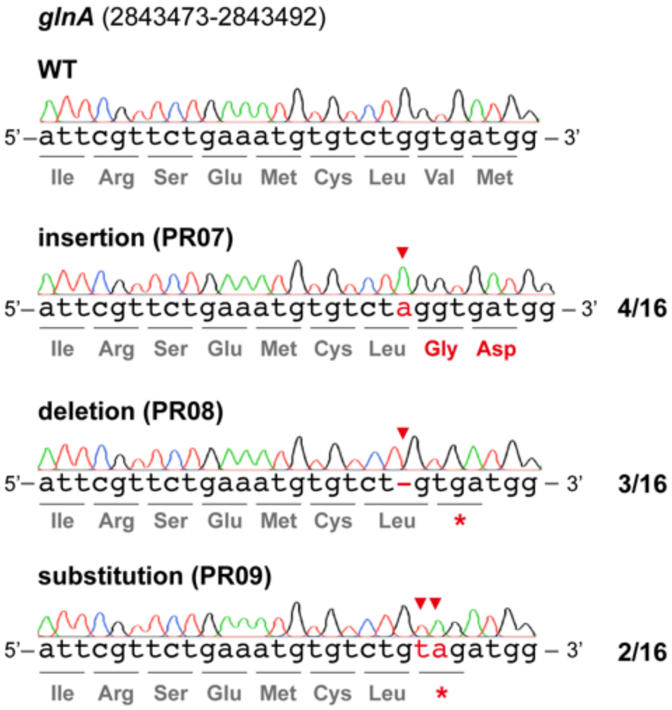

Figure S2. Prime editing results at a different locus in *glnA*. The editing was performed with pRC07, pRC08 and pRC09. The altered amino acids and the edited bases are highlighted in red, and the edited loci are indicated by the red arrows.

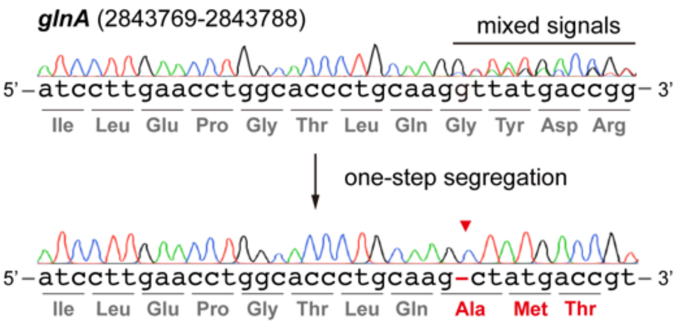

Figure S3. One-step segregation for pure edited strains. The altered amino acids and edited base are highlighted in red, and the edited position is indicated by the red arrow.

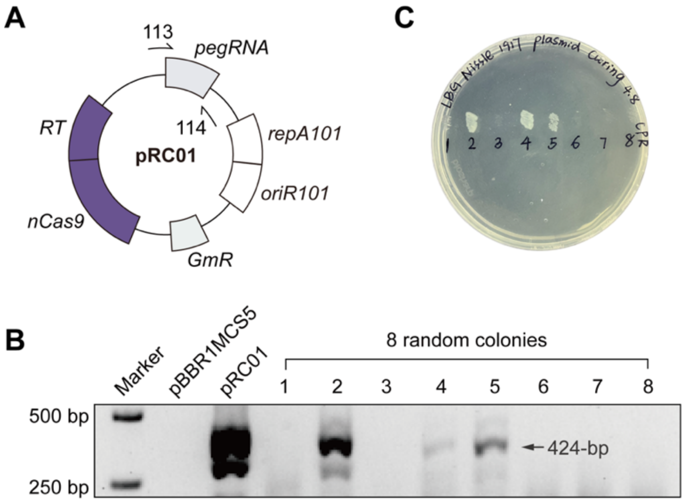

Figure S4. Plasmid curing of EcN. (A) Two primers, 113 and 114, were designed to amplify the specific sequences of pRC01 for verification. **(B)** Gel electrophoresis of plasmid cured and not cured colonies, with the backbone plasmid pBBR1MCS5 and pRC01 as negative and positive controls, respectively. **(C)** Sensitivity to gentamicin. The same colonies for gel electrophoresis were tested for gentamicin sensitivity. A colony with failed amplification and recovery sensitivity to gentamicin is considered plasmid cured.

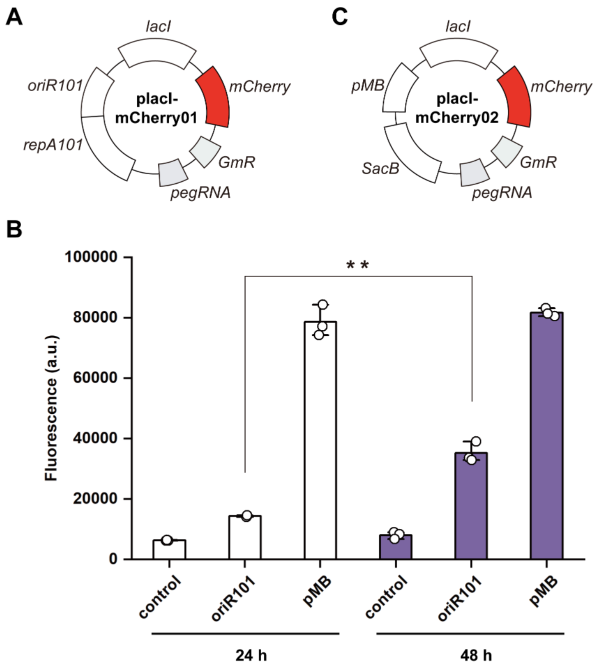

Figure S5. Verification of the increased expression levels with extended induction time. (A) The plasmid map of placI-mCherry01 and placI-mCherry02. **(B)** Fluorescence intensity. The fluorescence intensities for 24 h and 48 h induction are indicated in the white and purple columns, respectively. Three independent experiments were carried out and the standard deviations are represented by error bars.

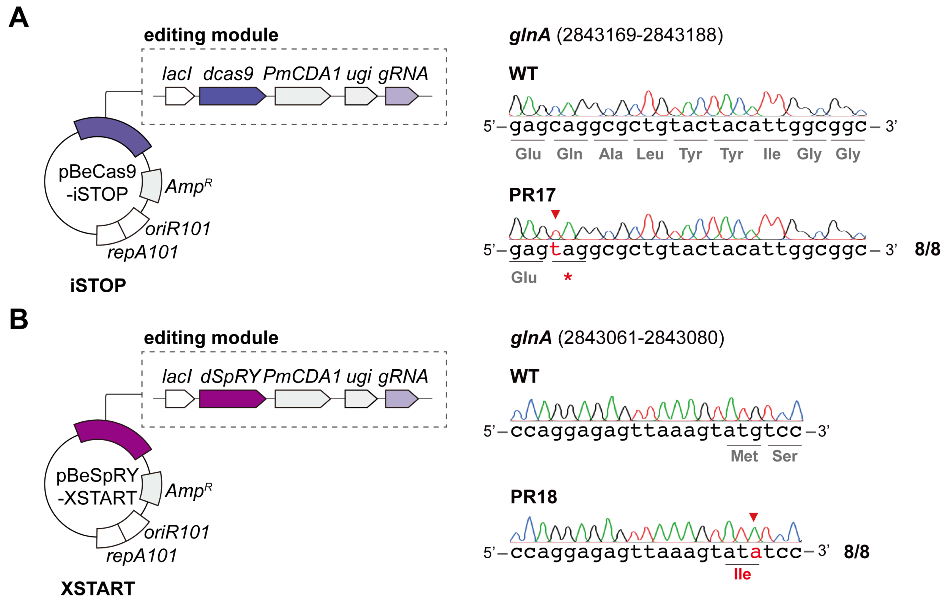

Figure S6. Base editing system for iSTOP and XSTRAT. (A) The plasmid map of pBeCas9-iSTOP and the sequencing result of PR17. The editing module contains *dCas9*, *PmCDA1* and *ugi*, driving by a *lacI*-P_{trc} inducible system. The *gRNA* is controlled by P_{J23119}. **(B)** The plasmid map of pBeSpRY-XSTART and the sequencing result of PR18. The editing module contains *dSpRY*, *PmCDA1* and *ugi*, driving by a *lacI*-P_{trc} inducible system. The *gRNA* is controlled by P_{J23119}. The altered amino acids and edited base are highlighted in red, and the edited position was indicated by the red arrow.

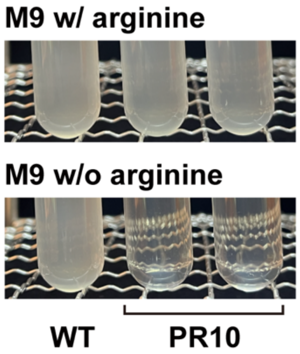

Figure S7. Growth of the barcoded auxotrophic PR10 in M9 minimal medium with and without arginine.

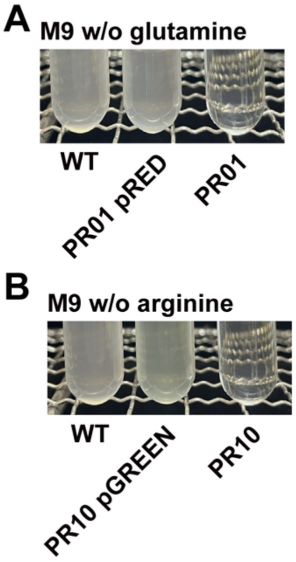

Figure S8. Phenotypical evaluation of the ARG-free platform. (A) Growth of PR01 with and without pRED in M9 medium without glutamine, and **(B)** growth of PR10 with and without pGREEN in M9 medium without arginine.

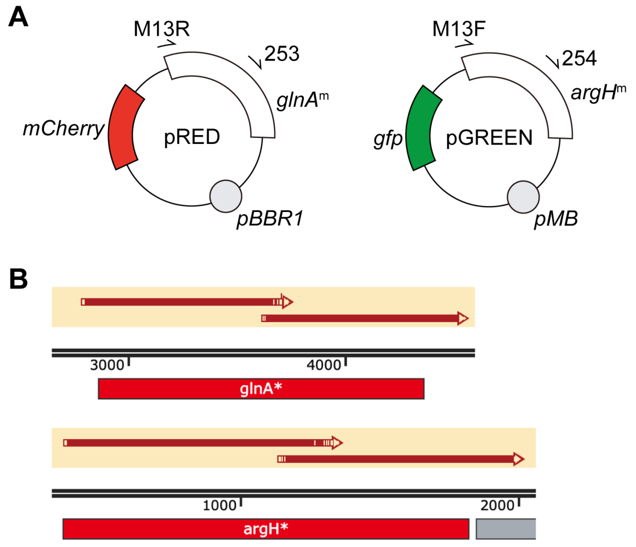

Figure S9. Design and construction of pRED and pGREEN. (A) Illustration of the two plasmids with sequencing primers highlighted on pRED (M13R and 253) and pGREEN (M13F and 254). **(C)** Sequencing verification of *glnA*^{m} and *argH*^{m} in pRED and pGREEN.

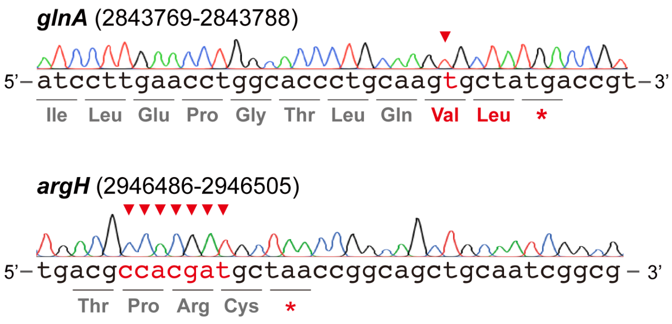

Figure S10. Sequencing results of the double-auxotrophic PR13. The altered amino acid and the edited base are highlighted in red, and the edited positions are indicated by red arrows.

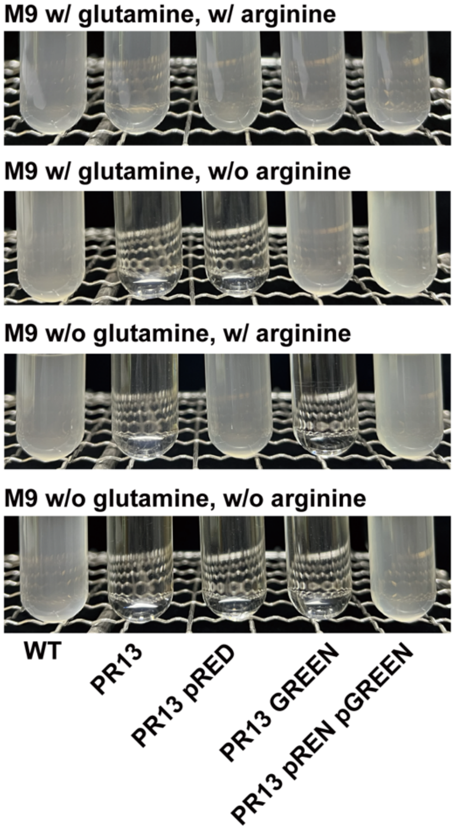

Figure S11. Phenotypical evaluation of the double-auxotrophic PR13 with pRED or/and pGREEN in M9 medium with and without glutamine and arginine.

Reference

1. Datsenko KA, Wanner BL. 2000. One-step inactivation of chromosomal genes in *Escherichia coli* K-12 using PCR products. Proc Natl Acad Sci U S A 97:6640-5.
2. Wei Y, Feng LJ, Yuan XZ, Wang SG, Xia PF. 2023. Developing a base editing system for marine *Roseobacter clade* bacteria. ACS Synth Biol 12:2178-2186.
3. Chen Y, Taton A, Go M, London RE, Pieper LM, Golden SS, Golden JW. 2016. Self-replicating shuttle vectors based on pANS, a small endogenous plasmid of the unicellular cyanobacterium *Synechococcus elongatus* PCC 7942. Microbiology (Reading) 162:2029-2041.
4. Anzalone AV, Randolph PB, Davis JR, Sousa AA, Koblan LW, Levy JM, Chen PJ, Wilson C, Newby GA, Raguram A, Liu DR. 2019. Search-and-replace genome editing without double-strand breaks or donor DNA. Nature 576:149-157.
5. Li X, Wei Y, Wang SY, Wang SG, Xia PF. 2024. One-for-all gene inactivation via PAM-independent base editing in bacteria. J Biol Chem doi:10.1016/j.jbc.2024.108113:108113.
